# Current Developments in Corneal Topography and Tomography

**DOI:** 10.3390/diagnostics11081466

**Published:** 2021-08-13

**Authors:** Piotr Kanclerz, Ramin Khoramnia, Xiaogang Wang

**Affiliations:** 1 Hygeia Clinic, Department of Ophthalmologyul, Jaśkowa Dolina 57, 80-286 Gdańsk, Poland; 2 Helsinki Retina Research Group, University of Helsinki, 00100 Helsinki, Finland; 3 The David J. Apple International Laboratory for Ocular Pathology, Department of Ophthalmology, University of Heidelberg, 69120 Heidelberg, Germany; ramin.khoramnia@med.uni-heidelberg.de; 4 Department of Cataract, Shanxi Eye Hospital, Taiyuan 030002, China; movie6521@163.com

**Keywords:** cornea, topography, tomography, optical coherence tomography, keratograph, scheimpflug imaging, pentacam

## Abstract

Introduction: Accurate assessment of the corneal shape is important in cataract and refractive surgery, both in screening of candidates as well as for analyzing postoperative outcomes. Although corneal topography and tomography are widely used, it is common that these technologies are confused. The aim of this study was to present the current developments of these technologies and particularly distinguish between corneal topography and tomography. Methods: The PubMed, Web of Science and Embase databases were the main resources used to investigate the medical literature. The following keywords were used in various combinations: cornea, corneal, topography, tomography, Scheimpflug, Pentacam, optical coherence tomography. Results: Topography is the study of the shape of the corneal surface, while tomography allows a three-dimensional section of the cornea to be presented. Corneal topographers can be divided into large- and small-cone Placido-based devices, as well as devices with color-LEDs. For corneal tomography, scanning slit or Scheimpflug imaging and optical coherence tomography may be employed. In several devices, corneal topography and tomography have been successfully combined with tear-film analysis, aberrometry, optical biometry and anterior/posterior segment optical coherence tomography. Conclusion: There is a wide variety of imaging techniques to obtain corneal power maps. As different technologies are used, it is imperative that doctors involved in corneal surgery understand the science and clinical application of devices for corneal evaluation in depth.

## 1. Introduction

Accurate assessment of the corneal shape is important in cataract and refractive surgery, both in screening of candidates for surgery as well as for analyzing postoperative outcomes [1,2,3]. It is also critical for the diagnosis of corneal disorders, which include keratoconus, pellucid marginal degeneration, corneal scars, limbal dermoid or pterygium. It can also be used in contact lens fitting, for assessment of intrastromal ring placement and several other conditions [4]. Primarily, corneal topography has only been used to describe the anterior surface of the cornea. Devices are now able to characterize both the anterior and posterior corneal surfaces, creating a three dimensional map of the cornea. Advances in digital photography and computer processing have immensely increased the utility of corneal imaging techniques.

It is imperative that doctors involved in corneal surgery understand the science and clinical application of devices for corneal evaluation in depth [4]. The aim of this study was to present the current developments of these technologies and particularly distinguish between corneal topography and tomography.

## 2. Methods

The PubMed, Web of Science and Embase databases were the main sources used to investigate the medical literature. An extensive search was conducted to identify articles in the matter of “corneal topography” and “corneal tomography” up to 28 June 2021 (Supplementary Materials). The following keywords were used in various combinations: cornea, corneal, topography, tomography, imaging, Scheimpflug, Pentacam, optical coherence tomography, OCT. Of the studies retrieved by this method, we reviewed all papers in English and the abstracts of non-English publications. The reference lists of these articles were also considered as a potential source of information. We attempted to present all methods that allowed a precise evaluation of the corneal shape. Emphasis was placed on studies published after the reviews by Oliveira et al. [5] and Shih et al. [6]. However, we aimed to present the current developments of these technologies and particularly distinguish corneal topography and tomography.

## 3. Results

The search identified 2633 unique articles. After removing duplicates and irrelevant studies, 91 articles were included in the review. Interestingly, a search of a combined phrase “topography” and “Pentacam”, which is obviously incorrect as Pentacam is a corneal tomographer, gave 687 results.

### 3.1. Corneal Topography

The expression topography is derived from the Greek words “place” (topos) and “to write” (graphein), which means to describe a place [7]. This was originally related to studying the shape of the Earth’s surface and features or those of planets, moons and asteroids [8]. Topography is the study of the shape of the corneal surface [9].

The beginnings of corneal topography date back to the 17th century [10]. A major advancement was achieved by António Plácido da Costa (1848–1915), a Portuguese ophthalmologist and microbiologist, who introduced a handheld device for precise evaluation of the corneal shape and published his report in 1880 [11,12,13]. The tool had a diameter of 23 cm, with painted concentric black and white circles, and an opening in the center of the device. The patient was to be placed in a well-lit location (e.g., in front of a window), and the corneal reflex from the keratoscope was to be evaluated at a distance of 15 cm from the cornea. Currently, most of the corneal topographers employ a Placido disc (the examination is historically named keratoscopy) and a system for image registration (videokeratoscopy).

**Figure 1:**
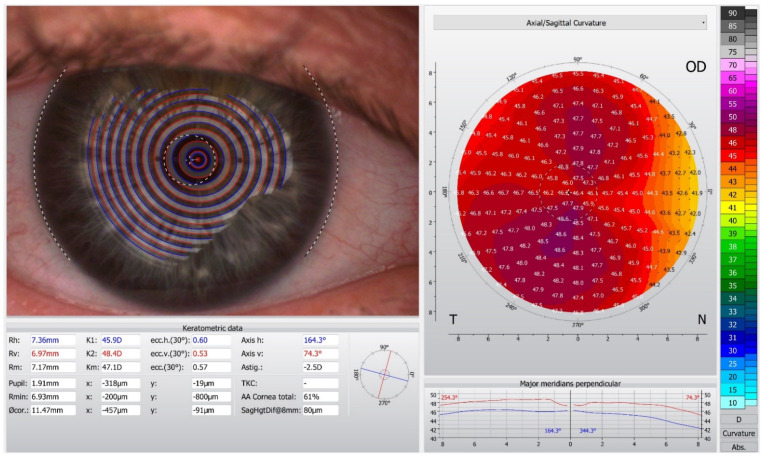
Corneal topography in the Oculus Keratograph 5 M.

The contemporary devices employed for corneal topography are presented in Table 1. Placido-disc devices can be classified as either large-cone (Figure 1) or small-cone systems. Small cones collect more data points and thus could be more accurate. However, they have a shorter working distance, which might make it more difficult to collect data in patients with deep orbits. Several Placido topographers acquire data based on 22 white Placido rings, with an angular resolution of 2 degrees [14]. Although corneal topographers allow instant image acquisition, their disadvantages include skew ray error [15,16], data interpolation at the corneal apex [17] and potential inaccuracy in areas of abrupt corneal elevation changes [18,19].

**Figure 2:**
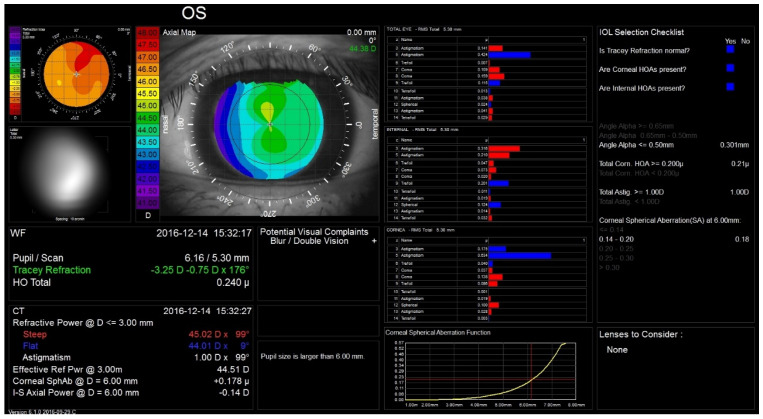
The Tracey iTrace device allows corneal aberrations to be analyzed.

Corneal topographers can also be used for the non-invasive assessment of the tear film; in this case, texture analysis of the Placido-ring is employed to detect tear film anomalies [20]. Additionally, an infrared ring illumination can be provided to prevent glare-related artifacts [21]. Currently, the Oculus Keratograph 5 M (K5 M; Oculus GmbH, Wetzlar, Germany) is one of the most commonly used tools to analyze the tear film [22]. The noninvasive keratograph tear break-up time readings were shown to display superior discriminative ability in detecting dry eye compared to conventional tear-film stability measurements [23]. Corneal topography has also been combined with aberrometry, e.g., in the iTrace (Tracey Technologies, Houston, TX, USA) and OPD-scan (Nidek CO. Ltd., Tokyo, Japan) [24,25]. In these devices, corneal topography and the wavefront map can be linked to each other, which enables subtracting of the corneal aberrations from the total eye aberrations (Figure 2). Moreover, the aforementioned devices provide repeatable measurements of the near and distance spherocylindrical refraction [26].

Topographical images can also be calculated by projecting other-than-Placido images on the corneal surface. The PAR Technology Corneal Topography System introduced by Belin et al. produced a true topographic map by analyzing a projected grid on the corneal surface [27,28,29]. Another modality of corneal topography, which is currently available commercially, is color-LED corneal topography. The method was introduced in 1997 [30], and the device was released commercially in the last few years. The Cassini (i-Optics, Hague, Netherlands) corneal topographer is able to analyze the corneal shape based on point-to-point reconstruction of specular reflections of 679 pseudo-random colored points [31]. The potential advantage of this approach over Placido-based systems is that it is not affected by the Placido mismatch, resulting in a proper reconstruction of non-rotationally symmetrical or distorted corneal surfaces [32,33,34]. In a study by Klijn et al., the magnitude of corneal astigmatism obtained with the Cassini topographer was not different to that obtained with the Pentacam (Oculus Optikgeräte, Wetzlar, Germany), the Lenstar (Haag-Streit, Koeniz, Switzerland) and the Keratotron (Optikon, Rome, Italy) [31]. With that, the repeatability of the cylinder measurements was higher than with the Pentacam or Keratotron (*p* < 0.001). Even though the keratometric values obtained with Cassini are similar to those of the Pentacam and IOL Master 500 (Carl Zeiss Meditec AG, Jena, Germany) [35,36] or the Orbscan IIz (Bausch and Lomb Surgical, Rochester, NY, USA) and Lenstar LS-900 (Haag-Streit Holding, Köniz, Switzerland) [37], the wide data spread discourages their interchangeable use to assess corneal power and astigmatism. Color LED topography also enables evaluation of the posterior surface using 2nd Purkinje imaging technology [38]. One might consider that measuring the total instead of anterior corneal astigmatism may decrease the residual astigmatism in toric IOL implantation [39]. The Cassini system has been shown to determine consistent measures of posterior corneal curvature and astigmatism in healthy eyes, but only measures of posterior astigmatism could be considered as interchangeable with those provided by the Pentacam [40]. In another study, for the astigmatism analysis, measurements from the anterior cornea obtained with color-LED topography showed an excellent agreement with Pentacam measurements, but the agreement was poor for the corneal posterior surface and particularly the magnitude of astigmatism [41,42]. Furthermore, when analyzing corneal aberrometry measurements obtained with the Cassini device in healthy eyes, they were not interchangeable with results provided by the Scheimpflug-based topography [43].

### 3.2. Corneal Tomography

Tomography is derived from the Greek words “cut section” (tomos) and “to write” (graphein). In medicine, the classic term computed tomography refers to a quickly rotating narrow X-ray beam, processed to generate cross-section images of an internal solid organ; based on these images, it is possible to produce a three-dimensional reconstruction of an anatomical structure. Similarly, corneal tomography allows the generation of a stereographic model of the cornea, enabling analysis of the front and back surfaces of the cornea, along with pachymetry mapping.

Currently, the corneal tomography images might be obtained with (i) Scanning slit devices, e.g., Orbscan IIz (Bausch & Lomb, Rochester, NY, USA); (ii) The Scheimpflug cameras, i.e., the Pentacam (OCULUS Optikgeräte GmbH, Wetzlar, Germany), Galilei (Ziemer Ophthalmic Systems AG, Port, Switzerland) and Sirius (CSO, Firenze, Italy); the latter two have an additional large cone Placido disc incorporated; (iii) OCT-based devices, e.g., the Anterion (Heidelberg Engineering, Heidelberg, Germany) Visante (Carl Zeiss Meditec AG, Jena, Germany) (Table 2).

Optical cross-sectioning for corneal analysis was first commercially introduced in 1995 with the Orbscan device (Bausch & Lomb Surgical, Rochester, NY, USA) [44,45]. The system employed slit scanning by a projection of 40 slits (12.50 mm high and 0.30 mm wide). The device calculated the corneal curvature based on the calculation of the front edge of the slits, but the images were not displayed for evaluation [45]. A significant problem was that the horizontal scanning did not have a shared point for the slits. Subsequently, the slit scanning system was combined with a Placido-disk attachment in the Orbscan II.

**Figure 3:**
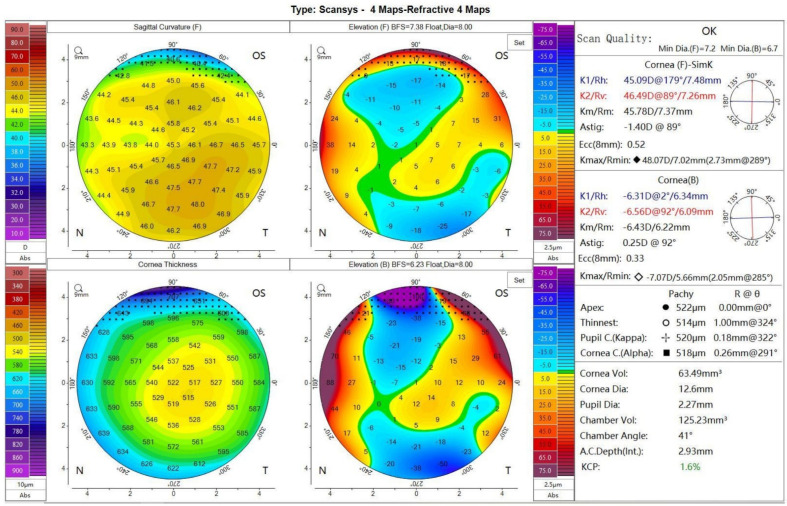
Scheimpflug corneal tomography in the Mediworks Scansys tomographer.

Digital Scheimpflug tomography has been recognized as the evolution of slit scanning systems [45] (Figure 3). Within these devices, a rotating Scheimpflug camera is employed; these systems have the ability to measure the dispersion of light along the optical axis, allowing the detection of changes in the transparency of the lens over time [46]. Devices with a rotating Scheimpflug camera evaluate not only the cornea, but the entire anterior segment from the anterior corneal surface to the posterior lens surface [47,48]. Visualizing the anterior chamber morphology is critical to establish the long-term safety of phakic IOLs. One of the most threatened potential complications of any type of anterior segment surgery, and particularly after anterior chamber and iris-fixated IOLs, is accelerated endothelial cell loss [49,50]. This risk has been shown to be negatively correlated with the anterior chamber depth, and the position of these IOLs in the anterior chamber is one of the main safety parameters in both preoperative screening and follow-up [51]. Assessment of anterior chamber morphology is also critical for implantable collamer lens (ICL) assessment; if an inserted ICL is too large, it might bow anteriorly, causing anterior chamber shallowing and introducing a risk of pupillary block and angle-closure glaucoma [52,53] In contrast, if the ICL vault is insufficient, it might potentially result in contact between the ICL and the crystalline lens, causing subsequent cataract formation [54].

**Figure 4:**
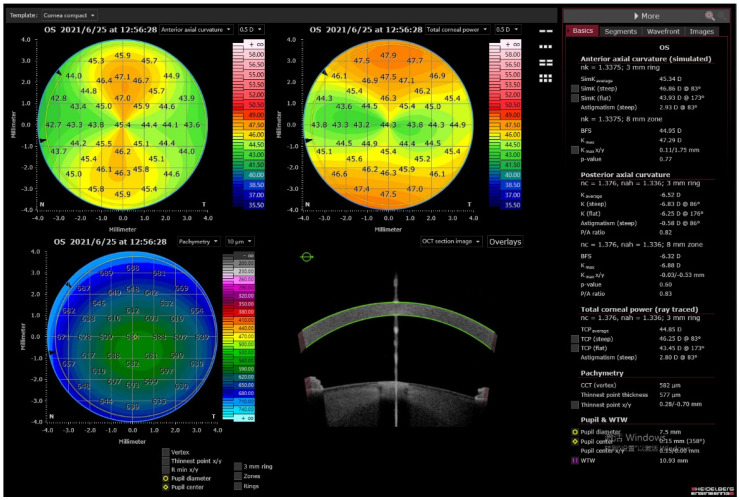
Corneal tomography in a swept-source optical coherence tomography device, the Anterion (Heidelberg Engineering).

Corneal tomography characterizes the elevation of the front and back corneal surfaces and reconstructs the pachymetric mapping, which has significantly enhanced the sensitivity and specificity for detecting corneal ectasia [8,55]. A significant advantage of tomography compared to topography is the possibility to determine the true corneal power; to calculate it, it is required to assess the posterior corneal surface. In most keratometric devices, the relationship between the anterior and posterior corneal surfaces is considered as constant and estimated based on a theoretical “keratometric index”. Evaluation of the power of the posterior corneal surface is critical in IOL calculations in eyes having undergone laser vision correction. As in corneal refractive surgery, corneal tissue is removed for refractive purposes, and as a consequence, the altered relationship between the front and back surfaces invalidates the use of the standardized index of refraction. Moreover, recent investigations showed that in virgin eyes, the magnitude of anterior and posterior astigmatism is greater when the steep axis of the anterior astigmatism is oriented vertically [56]. Thus, neglecting measurements of the posterior corneal surface might result in overestimation of with-the-rule astigmatism, whereas in eyes with against-the-rule astigmatism, the magnitude of astigmatism can easily be underestimated. Therefore, accurate assessment of the total corneal power, and specifically its astigmatism, with corneal tomography devices could potentially increase the refractive outcome in cataract and refractive lens extraction surgery [57,58,59,60]. Currently, the Zeiss IOL Master 700, Oculus Pentacam AXL and Anterion (Heidelberg Engineering, Heidelberg, Germany) allow measurement of the posterior corneal astigmatism [56] (Figure 4). Moreover, an ultra-fast Scheimpflug camera was implemented in the Corvis ST (OCULUS Optikgeräte GmbH, Wetzlar, Germany). Corvis ST is a non-contact tonometer, which allows information about the biomechanical properties of the cornea to be obtained by tomographical assessment of the deformation caused by the air stream directed at the eye [61,62].

Optical coherence tomography (OCT) systems analyze measurements of the echo time delay of backscattered or backreflected light by using an interferometer with a mechanically scanned optical reference path [63,64,65]. OCT devices can be classified into spectral-domain OCT (SD-OCT) and time-domain OCT (TD-OCT). SD-OCT is associated with a rapid scan speed, less noise and higher resolution compared to TD-OCT, but the imaging field is smaller [66]. Swept-source OCT (SS-OCT) devices use a short-cavity swept laser instead of the superluminescent diode laser typical for conventional SD-OCT [67]. The shorter the wavelength used in the OCT device (Table 2), the shorter is the imaging range. Both SD-OCT and TD-OCT allow visualization of the cornea, anterior chamber and iridocorneal angle [66,68,69,70]. The applicability of employing OCT for corneal tomography was demonstrated more than 10 years ago [71,72]. A problem in obtaining corneal tomography images with OCT is the fan distortion [71]; thus, some SD-OCT devices allow only pachymetry but not tomography images to be obtained. For example, the Optovue SD-OCT (Freemont, CA, USA) is able to acquire eight evenly spaced 6.0 mm radial cross-sections in order to provide corneal curvature data and corneal and epithelial thickness maps; however, it does not allow for the obtaining of precise maps of the corneal power [73,74]. In the Zeiss Visante OMNI platform, the OCT results are combined with those from a Placido-ring topography, to calculate a three-dimensional model [75]. Some other OCT devices, e.g., the Casia SS-1000 and Casia 2 (Tomey, Nürnberg, Germany) or Optopol Revo (Optopol Technology Sp. z o.o., Zawiercie, Poland), are able to calculate posterior corneal surface power and elevation, as in Scheimpflug imaging, but without topographic data. Gjerdrum et al. have shown that OCT devices, in particular the Casia, might have a greater variability in simulated keratometry values than the Pentacam [76]. Szalai et al. have shown the utility of SD-OCT (Casia SS-1000) in measurements of eyes with keratoconus; although the results for keratometry, pachymetry and anterior chamber depth with Casia SS-1000 were different to those obtained with Pentacam, but the repeatability was similar [77]. Similarly, as with Scheimpflug cameras, OCT devices allow precise imaging of the anterior chamber, which is critical for, e.g., phakic IOLs or ICL assessment. One of the main limitations of Scheimpflug tomography is the low resolution and poor image quality; in these terms, OCT devices allow significantly better quality images with higher definition to be obtained [78]. OCT allows the corneal epithelium to be visualized, which, in certain conditions, might manifest as local thinning (e.g., in keratoconus) or thickening (e.g., adjacent to a corneal scar); this is an advantage over corneal topography, which presents solely the morphology of the corneal surface [79].

## 4. Discussion

### 4.1. Confusion in Terminology

Although corneal topography and tomography are widely used, it is common that these technologies are confused [4,6,41,80,81,82,83,84]. This corrigendum could be associated with the fact that at the time when corneal tomographers were developed, the nomenclature was not yet defined [8]. The original Orbscan systems were designed to provide a three-dimensional reconstruction (tomography) of the cornea; however, the measurements were referred to as ‘topography’ (Orbscan topography system). The current version of the Orbscan device (Orbscan III) is referred to as an anterior segment analyzer but also as a multidimensional Orbscan topographer. Still, the Orbscan III displays Placido-discs but in a modified form and should rather be classified as a tomographer than topographer. Furthermore, the corneal module for Casia 2 (Tomey, Nürnberg, Germany) is advertised as corneal topography, although it does not have a Placido-cone and technically is an OCT corneal tomographer. Distinguishing topography and tomography is critical, as each of these examinations have their own characteristics. For corneal topography, we can expect excellent agreement in corneal power between measurements; for tomography the agreement limits are wider [85]. On the other hand, tomography allows critical stereometric data to be obtained, which are not available in corneal topography.

### 4.2. Limitations of Current Techniques

Both corneal topography and tomography are non-invasive measurements and carry no risk for the patient. However, they do require the patient to maintain a fixed gaze and can be inaccurate with eye movement. For Scheimpflug devices, it takes 2.0 s to obtain 25–50 scans as the camera rotates around the eye. Potential eye movements during the two-second scan can occur; two cameras (e.g., in the Ziemer Galilei) allow measurements to be averaged and minimize decentration, such as in the case of involuntary eye movement [86]. On the contrary, Placido disk devices provide an instant picture of the cornea. It is known that Scheimpflug devices using a rotating camera can allow accurate measurements from highly irregular corneas that reflective Placido-based systems struggle to represent accurately. However, the distortion of the camera optics and of the cornea and lens itself distort the image, requiring automatic distortion correction [87]. Slit-scanning machines are imprecise when assessing the posterior surface after corneal refractive surgery due to the disruption of the corneal interface, causing light scatter [88,89,90].

Scheimpflug devices also provide a good-looking image of the anterior segment in some particular meridian. However, this is just a reconstruction but not a true image of the corneal surface. Moreover, the true net power and keratometric power deviation have limited physical value outside of the central 2 to 3 mm. As Scheimpflug systems employ 470–475 nm wavelength light, they are sensitive to corneal opacities, resulting in hyperreflective images of an inaccurate contour [46]. Due to total internal reflection in the peripheral cornea, direct visualization of the anterior chamber angle is not possible. However, the extrapolation software is able to provide an estimate of the iris–corneal angle with relatively high accuracy [91]. OCT could be considered more practical for evaluating the peripheral corneal and corneo-scleral region [92]. OCT is particularly useful for a contact lens practitioner during both contact lens fitting and assessment, as the interaction of the lens and the cornea, as well as the edge with the conjunctiva, can be quantitatively assessed [92].

### 4.3. Future Developments

Currently, we are encountering a significant development of optical technologies; they exhibit faster scanning speed and employ more reliable tracking systems. Still, conventional Placido-ring topography might provide the most realistic projection of the corneal surface. It is therefore unlikely that classical corneal topography will be completely replaced by corneal tomography.

In the future, combining technologies in order to create more versatile devices could be a viable option (Table 3). In order to prevent refractive surprises and improve the predictability of intraocular lens power calculation, corneal topography has been employed in some optical biometers, e.g., the Aladdin (Topcon Corporation, Tokyo, Japan), or is available as an option in others, e.g., the Lenstar LS-900 (Haag Streit AG, Bern, Switzerland). On the contrary, optical biometry has been added to the new models of corneal tomography devices by adding a partial coherence interferometry SLED diode (e.g., Pentacam AXL) or low-coherence optical reflectometry with a superluminescent diode laser (e.g., Ziemer Galilei G6). Furthermore, a corneal tomography function has been added to a commercially available anterior/posterior segment SD-OCT device (the Revo NX, Optopol Technology Sp. z o.o., Zawiercie, Poland) [82]. Many of these combinations would be considered practical, as with technological development, the number of devices required to provide a satisfactory ophthalmological standard of care has increased in recent decades.

## 5. Conclusions

There is a wide variety of imaging techniques to obtain corneal power maps. As different technologies are used, it is imperative that doctors involved in corneal surgery understand the science and clinical application of devices for corneal evaluation in depth. Advances in digital photography and computer processing have immensely increased the utility of corneal topography and tomography.

## Figures and Tables

**Table 1:** Techniques employed for corneal topography in some of the currently used devices.

Technology	Details	Example Topographers
**Placido disc**	Large-cone topography	CSO Antares, CSO Sirius+ *, CSO MS-39 * Oculus Keratograph 5 M Topcon KR-1W Zeiss Atlas Ziemer Galilei *
**Placido disc**	Small-cone topography	Medmont E300 Optikon Keratotron
**Color light-emitting diode**	Point-to-point reconstruction of specular reflections	i-Optics Cassini i-Optics Cassini Ambient

* Devices that enable both corneal topography and tomography images to be obtained.

**Table 2:** Techniques employed for corneal tomography in some of the currently used devices.

Technology	Light Source (Wavelength)	Example Tomographers
Scanning slit	white flash light	Orbscan II
Scheimpflug imaging	blue-light emitting diode (470–475 nm)	CSO Sirius+ * Mediworks Scansys Oculus Pentacam Ziemer Galilei *
OCT	superluminescent diode laser (830–845 nm)	CSO MS-39 * Optopol Revo
SS-OCT	rapidly tuned laser with longer wavelength (1310 nm)	Heidelberg Engineering Anterion Tomey Casia SS-1000/Casia 2 Zeiss Visante OMNI *

* Devices that enable both corneal topography and tomography images to be obtained. Abbreviations: LCOR—low-coherence optical reflectometry, OCT—optical coherence tomography, PCI—partial coherence interferometry, SS-OCT—swept source-OCT.

**Table 3:** Devices combining topography or tomography with other technologies.

Corneal Topography + Ocular Aberrometry	Nidek OPD-Scan Tracey iTrace
Corneal topography + ocular biometry	Topcon Aladdin Lenstar LS-900
Corneal tomography + ocular biometry	Pentacam AXL Ziemer Galilei G6 Heidelberg Engineering Anterion
Corneal tomography + ocular biometry + posterior segment optical coherence tomography	Optopol Revo NX

## Supplementary Materials

The following are available online at https://www.mdpi.com/article/10.3390/diagnostics11081466/s1, Supplement 1: Search strategy.
