# Supplementary material for: Current Developments in Corneal Topography and Tomography
Source: Diagnostics (Basel). 2021 Aug 13;11(8):1466. doi: 10.3390/diagnostics11081466 (PMC8392046; doi:10.3390/diagnostics11081466)
Supplement: Supplementary file 1 [file diagnostics-11-01466-s001.zip › diagnostics-1326518-supplementary.pdf]

Supplement 1. Search strategy

Literature searches of the PubMed and Web of Science databases were conducted on June 28, 2021; the search strategies are as follows. Specific limited update searches were conducted after June 28, 2021. Reference lists of the included studies were also considered as a source of publications.

A.1. PubMed Search (Publication Date 1/10/11–06/28/2021)
((cornea[Title]) OR (corneal[Title])) AND ((imaging[Title]) OR (tomography[Title]) OR (topography[Title]) OR (Scheimpflug[Title]) OR (Pentacam[Title]) OR ("optical coherence tomography"[Title]) OR (OCT[Title]))
1907 references.

A.2. Web Of Science Search (Publication Date 1/10/11–06/28/2021)
(TI=("cornea") OR TI=("corneal")) AND (TI=("imaging") OR TI=("tomography") OR TI=("Scheimpflug") OR TI=("Pentacam") OR TI=("optical coherence tomography") OR TI=("OCT"))
Indexes=SCI-EXPANDED, SSCI, A&HCI, CPCI-S, CPCI-SSH, BKCI-S, BKCI-SSH, ESCI, CCR-EXPANDED, IC
Timespan=All years
1386 references.

A.3. PubMed Search (Publication Date 1/10/11–06/28/2021)
Search: pentacam and topography Sort by: Most Recent
pentacam: "pentacam"[All Fields] AND "pentacam's"[All Fields]
topography: "topographies"[All Fields] AND "topography"[All Fields]
675 references

A.4. Web Of Science Search (Publication Date 1/10/11–06/28/2021)
(AB=(pentacam)) AND AB=(topography)
Indexes=SCI-EXPANDED, SSCI, A&HCI, CPCI-S, CPCI-SSH, BKCI-S, BKCI-SSH, ESCI, CCR-EXPANDED, IC
Timespan=All years
255 references.
